# Associations Between Nutritional Factors, Obesity and Ocular Diseases: A Narrative Literature Review

**DOI:** 10.3390/nu17233798

**Published:** 2025-12-03

**Authors:** Corina Georgiana Bogdănici, Camelia Margareta Bogdănici, Irina Andreea Pavel, Cosmin Victor Ganea, Vlad Constantin Donica, Elena Mihaela Cărăușu

**Affiliations:** 1 Doctoral School, Grigore T. Popa University of Medicine and Pharmacy, University Street, No. 16, 700115 Iasi, Romania; bogdanici_corina-georgiana@d.umfiasi.ro (C.G.B.); cosmin-victor.ganea@umfiasi.ro (C.V.G.); 2 Department of Ophthalmology, Faculty of Medicine, Grigore T. Popa University of Medicine and Pharmacy, University Street, No. 16, 700115 Iasi, Romania; camelia.bogdanici@umfiasi.ro (C.M.B.); vlad-constantin.donica@d.umfiasi.ro (V.C.D.); 3 Department of Health Management, Faculty of Dental Medicine, Grigore T. Popa University of Medicine and Pharmacy, University Street, No. 16, 700115 Iasi, Romania; elena.carausu@umfiasi.ro

**Keywords:** micronutrients, vitamin deficiency, mineral deficiency, ocular health, diet, oxidative stress, inflammation

## Abstract

**Background**: Nutritional imbalances significantly affect ocular physiology, contributing to dry eye disease, cataracts, age-related macular degeneration (AMD), and optic neuropathies. This review summarizes recent evidence on how micronutrient deficiencies and obesity influence eye health. **Methods**: A narrative search was performed in PubMed, Scopus, and ScienceDirect (last 10 years). Human studies evaluating associations between micronutrients, dietary patterns, obesity, and ocular diseases were included. Out of 843 records, 50 studies met the eligibility criteria. **Results**: Deficiencies in vitamins A, D, E, C, and B-complex were consistently linked to ocular surface inflammation, retinal oxidative stress, cataracts, AMD, and nutritional optic neuropathies. Altered levels of zinc, copper, selenium, and magnesium were associated with impaired photoreceptor function, glaucoma risk, and retinal degeneration. Obesity emerged as an independent risk factor for AMD, diabetic retinopathy, and glaucoma through mechanisms involving oxidative stress and vascular dysfunction. Evidence from AREDS/AREDS2 supports targeted antioxidant supplementation in intermediate AMD. **Conclusions**: Adequate nutritional status and metabolic balance play a critical role in preserving ocular health. Early detection and correction of deficiencies may prevent or slow the progression of several eye diseases. Further high-quality trials are needed to define optimal nutritional recommendations.

## 1. Introduction

The human eye is among the most metabolically active organs, consuming oxygen at a rate comparable to that of the brain and myocardium. This high metabolic demand makes it particularly vulnerable to nutritional deficiencies and oxidative damage. The retina, in particular, is composed of specialized neurons and photoreceptors that require a continuous supply of micronutrients, including vitamins A, C, E, B-complex, and trace elements like zinc and selenium, to support phototransduction, maintain cellular membrane integrity, and modulate inflammation [1].

Micronutrients play diverse roles in ocular health, such as acting as antioxidants, enzymatic cofactors, and immune regulators. They help prevent lipid peroxidation, DNA damage, and vascular dysfunction, processes that are strongly implicated in the pathogenesis of major ocular diseases. Vitamin A, for instance, is essential for the synthesis of rhodopsin, a key protein in scotopic vision. Vitamin C stabilizes collagen fibers in the cornea and sclera, while zinc is involved in the regeneration of visual pigments in the photoreceptors [2].

In recent decades, shifting dietary patterns, particularly increased consumption of ultra-processed foods and reduced intake of fruits, vegetables, and nutrient-dense whole foods, have led to subclinical or overt micronutrient deficiencies. Combined with the rising prevalence of obesity, these dietary changes are now recognized as major contributors to the onset and progression of multiple eye conditions, including xerophthalmia, cataracts, age-related macular degeneration (AMD), diabetic retinopathy, and optic neuropathies [3].

Recent data published in 2025 further reinforce the link between nutritional status and ocular disease. A large population-based analysis identified an optimal serum 25(OH)D threshold around 50 nmol/L for reducing the risk of cataract, AMD and diabetic retinopathy, suggesting a clinically relevant vitamin D target for eye health [4]. Complementary Mendelian randomization work has provided additional evidence for potential causal relationships between circulating 25(OH)D levels and several ocular disorders, including AMD, cataract, diabetic retinopathy and glaucoma [5]. Recent nutritional and dietary studies also indicate that high-fat, pro-inflammatory diets increase the risk of ocular disease through oxidative stress and lipid dysregulation, whereas Mediterranean-style or antioxidant-rich dietary patterns appear protective for retinal structure and function [6,7]. Together, these findings highlight the need for an updated synthesis of how micronutrient status and diet-related metabolic disturbances influence ocular health and disease.

## 2. Materials and Methods

We searched PubMed, Scopus and Science Direct databases, focusing on studies published within the last 10 years. Search filters included English language and human clinical studies. Searches were conducted between February and March, 2025. We used the following keywords: micronutrients, vitamin deficiency, mineral deficiency, vision, diet, eye health, obesity, to find relevant articles on the selected topic. The search used combinations of the following keywords and the Boolean operators AND/OR: micronutrients, vitamin deficiency, mineral deficiency, vision, diet, eye health, obesity, retina, cornea, age-related macular degeneration, glaucoma, diabetic retinopathy, cataract, dry eye disease. Inclusion criteria: human studies (randomized controlled trials, cohort, case–control, cross-sectional), systematic reviews, and meta-analyses evaluating associations between nutritional factors or obesity and ocular diseases such as AMD, diabetic retinopathy, glaucoma, cataract, dry eye disease, and optic neuropathies. Exclusion criteria: non-English publications, conference abstracts, editorials, letters, protocols, preprints, or purely surgical/animal/in vitro studies without human clinical relevance.

From each included study, data were extracted regarding the study design, population, nutritional exposure (specific micronutrient, dietary pattern, or obesity metric), ocular outcome, and key findings. The quality and strength of the available evidence were appraised based on study design and consistency of results. Highest weight was given to meta-analyses and randomized controlled trials, followed by prospective cohort and case–control studies, while cross-sectional, mechanistic, or experimental data were used mainly to support biological plausibility. Intermediate exclusion numbers were not consistently available across all databases; therefore, only final counts are reported.

**Figure 1:**
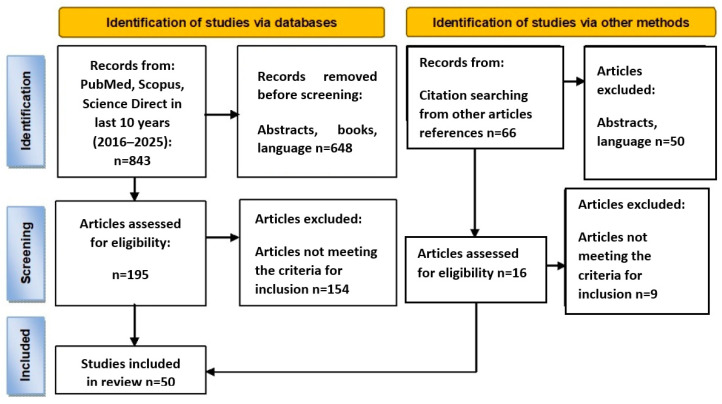
PRISMA diagram illustrating the literature search and selection process.

Our search resulted in 843 total references, and 50 papers were within our scope of interest. The selection process is illustrated using the PRISMA 2020 flow diagram [8] (Figure 1).

## 3. Vitamins and Minerals Deficiencies

### 3.1. Vitamin A Deficiency

Vitamin A plays a pivotal role in maintaining the health of the ocular surface, epithelial integrity, mucin production, and photoreceptor function. Retinol and its derivatives are essential for the synthesis of rhodopsin, the visual pigment required for low-light (scotopic) vision. A deficiency in vitamin A disrupts this visual cycle, leading to early symptoms such as night blindness (nyctalopia) [9].

With progression, more severe signs occur, including xerophthalmia, conjunctival xerosis, Bitot’s spots, corneal xerosis, and keratomalacia, which, if left untreated, can result in irreversible blindness [10]. These conditions remain widespread in developing regions, especially among malnourished children, with vitamin A deficiency ranking as a leading cause of preventable childhood blindness globally. Beyond phototransduction, retinoids regulate the differentiation and function of epithelial cells of the conjunctiva and cornea, and modulate local immune responses, crucial in protecting the ocular surface from infections and desiccation. Vitamin A may enhance mucin gene expression in goblet cells and support tear film stability [11]. Recent studies highlight link between subclinical vitamin A deficiency and dry eye disease, meibomian gland a dysfunction, and increased susceptibility to ocular infections [12]. In at-risk populations, particularly in Sub-Saharan Africa and Southeast Asia, large-scale supplementation programs and food fortification (e.g., biofortified rice and oil) have significantly reduced the incidence of corneal ulcers and blindness. Therapeutic intervention with high-dose vitamin A remains the gold standard for reversing early ocular symptoms and preventing permanent vision loss [13].

### 3.2. Vitamin D Deficiency

Vitamin D is increasingly recognized for its immunomodulatory, anti-inflammatory, and neuroprotective roles in ocular tissues. The biologically active form, calcitriol (1,25-dihydroxyvitamin D), binds to vitamin D receptors (VDRs) expressed in the cornea, conjunctiva, retina, and lacrimal glands. Through these pathways, it modulates local inflammation, maintains epithelial integrity, and regulates tear secretion and meibomian gland function [14].

Deficiency in vitamin D has been linked with a higher prevalence and severity of dry eye disease (DED), particularly in postmenopausal women and patients with autoimmune conditions. Studies have demonstrated that lower serum 25(OH)D levels correlate with increased ocular surface staining, tear film instability, and elevated inflammatory markers such as IL-1 and TNF-α in the conjunctiva [15].

In addition, vitamin D deficiency has been associated with a greater risk of posterior subcapsular cataracts, diabetic retinopathy, and even age-related macular degeneration (AMD), possibly due to its association with oxidative stress modulation, vascular endothelial stability, and inhibition of neovascularization [16].

### 3.3. Vitamin E Deficiency

Vitamin E, a potent lipid-soluble antioxidant, is critical for neutralizing reactive oxygen species (ROS) within retinal tissues, especially in the photoreceptor outer segments, which contain high concentrations of polyunsaturated fatty acids. These photoreceptors are particularly susceptible to oxidative stress, which plays a central role in the pathogenesis of age-related macular degeneration (AMD), retinitis pigmentosa, and other degenerative retinal diseases [3].

Deficiency in vitamin E can result in retinal pigment epithelium (RPE) dysfunction, photoreceptor apoptosis, and progressive retinal degeneration. Clinical findings in vitamin E–deficient individuals include reduced contrast sensitivity, nyctalopia, and peripheral visual field constriction, closely mimicking retinitis pigmentosa in severe cases [17].

Experimental models suggest that alpha-tocopherol may protect retinal neurons and helps preserve mitochondrial function and limit oxidative-induced lipid peroxidation. In patients with inherited vitamin E malabsorption syndromes, long-term deficiency often leads to irreversible vision loss unless corrected early [18].

Furthermore, several studies support the role of vitamin E supplementation in slowing the progression of AMD. In the Age-Related Eye Disease Study (AREDS), vitamin E, combined with vitamin C, beta-carotene, and zinc, significantly reduced the risk of disease progression in patients with intermediate or advanced AMD [19]. More recent meta-analyses suggest that alpha-tocopherol supplementation improves oxidative stress markers and may enhance functional outcomes in diabetic retinopathy and glaucoma, though results vary depending on dosage and baseline status [20].

### 3.4. Vitamin B-Complex Deficiencies

B-complex vitamins play a vital role in maintaining neuroretinal function, vascular health, mitochondrial integrity, and neurotransmitter synthesis. Given the eye’s high metabolic rate and its dependence on precise neurosensory transmission, even marginal deficiencies in B vitamins can result in profound ocular dysfunction. Thiamine (Vitamin B1) is necessary for energy production in neural tissues. Deficiency is classically associated with Wernicke’s encephalopathy, which includes a triad of nystagmus, ophthalmoplegia, and mental confusion. These signs are particularly seen in chronic alcoholics and patients with malnutrition or prolonged parenteral nutrition [21].

Riboflavin (Vitamin B2) is a coenzyme in redox reactions and supports glutathione regeneration. Its deficiency may result in keratoconjunctivitis, corneal vascularization, and photophobia, particularly in populations with restricted diets or malabsorption syndromes. Riboflavin also enhances corneal collagen cross-linking, a therapy used in keratoconus [22].

Pyridoxine (Vitamin B6) and cobalamin (Vitamin B12) are critical for myelin synthesis and neuronal health. Deficiencies in these vitamins are linked to bilateral optic neuropathies, presenting with progressive central vision loss, color desaturation, and central or cecocentral scotomas. These manifestations are common in vegans, elderly individuals, and patients with gastrointestinal disorders or chronic alcoholism [23].

Folate (Vitamin B9), while primarily known for its role in hematopoiesis, also supports retinal metabolism, DNA synthesis, and endothelial function. Low folate levels may exacerbate vascular compromise in diabetic retinopathy and contribute to homocysteine-mediated oxidative stress in retinal capillaries [24].

### 3.5. Vitamin C Deficiency

Vitamin C (ascorbic acid) is a powerful water-soluble antioxidant essential for collagen biosynthesis, tissue repair, and protection against oxidative damage. In the eye, it contributes significantly to maintaining the structural integrity of the sclera, cornea, and conjunctival vasculature, where it facilitates hydroxylation of proline and lysine residues critical to collagen formation [25,26].

Deficiency of vitamin C leads to weakened capillary walls and extracellular matrix disruption, manifesting clinically as subconjunctival hemorrhages, delayed corneal wound healing, and increased vascular fragility. In severe cases, particularly in elderly or malnourished individuals, scurvy may present with ocular bleeding, lid ecchymosis, and conjunctival pallor [27,28].

Oxidative stress is a key driver in the development of cataracts, and vitamin C plays a protective role by neutralizing reactive oxygen species in the aqueous humor and lens fibers. Decreased ascorbic acid levels in the aqueous humor have been consistently documented in patients with senile nuclear and cortical cataracts, indicating its protective role in lens transparency [29,30,31].

Additionally, experimental studies have shown that vitamin C supplementation may delay cataractogenesis in diabetic models and may support epithelial barrier regeneration in dry eye disease, though human trial data remain limited [32].

### 3.6. Zinc and Copper Deficiencies

Zinc is a trace mineral critical for ocular physiology, particularly within the retina and retinal pigment epithelium (RPE). It functions as a cofactor for retinal dehydrogenase, which is essential in the conversion of retinol to retinal in the visual cycle. Moreover, zinc stabilizes cellular membranes, supports antioxidant enzymes like superoxide dismutase (SOD), and modulates apoptosis in retinal cells [33].

Zinc deficiency has been associated with night blindness, due to its impairment of rhodopsin regeneration. It also leads to delayed corneal and conjunctival wound healing, increased oxidative stress, and impaired immune response at the ocular surface [34]. Chronic low zinc status has been associated with AMD development, possibly through increased retinal oxidative damage and impaired DNA repair mechanisms. The landmark Age-Related Eye Disease Study (AREDS) demonstrated that high-dose zinc supplementation, in combination with antioxidants, slowed the progression of intermediate to advanced AMD by 25% in a 5-year period [19,35]. Subsequent analysis suggests that zinc’s benefit may depend on genotype and dosage balance with copper to prevent hypocupremia [36].

Copper is a trace element involved in mitochondrial respiration, antioxidant defense (as a cofactor of superoxide dismutase), and iron metabolism. In the visual system, copper is essential for maintaining optic nerve integrity and myelin synthesis. Copper deficiency has been associated with progressive optic neuropathy, clinically resembling vitamin B12 deficiency, and is often characterized by bilateral vision loss, central or cecocentral scotomas, and optic disc pallor. This condition has been increasingly reported in patients on prolonged zinc supplementation, post-gastric bypass, or with malabsorption syndromes. Prompt recognition is crucial, as optic nerve damage may become irreversible if untreated [37,38,39].

### 3.7. Selenium and Magnesium Deficiencies

Selenium is a trace antioxidant mineral that plays a central role in the glutathione peroxidase (GPx) enzyme system, which protects retinal tissues from oxidative stress. It acts synergistically with vitamin E in neutralizing lipid peroxides and maintaining membrane integrity, especially within the photoreceptor layer of the retina. Selenium deficiency has been associated with increased susceptibility to retinal degeneration, cataractogenesis, and impaired repair of oxidative DNA lesions. Animal models demonstrate that insufficient selenium leads to apoptosis in the retinal pigment epithelium and accelerated cataract formation, particularly under conditions of light exposure or diabetic oxidative stress [40,41,42].

Magnesium, an essential cofactor in over 300 enzymatic reactions, is critical for vascular tone regulation, endothelial integrity, and neurotransmission. In the eye, magnesium contributes to the autoregulation of retinal blood flow and protection against ischemia–reperfusion injury. Multiple observational studies have linked low serum magnesium levels to increased risk of diabetic retinopathy, likely through its influence on insulin sensitivity, oxidative stress, and microvascular integrity. Moreover, magnesium deficiency has been implicated in glaucoma pathophysiology, potentially due to its effects on trabecular meshwork tone and optic nerve perfusion. Some pilot trials suggest magnesium supplementation may help reduce intraocular pressure and improve visual field stability in early glaucoma [43,44,45]. Animal experimental results are mentioned only to illustrate underlying biological mechanisms, as their applicability to human ocular disease is limited and clinical findings remain the primary source of interpretation.

## 4. Ocular Implications of Malabsorption Syndromes

Malabsorption syndromes such as celiac disease, inflammatory bowel disease (IBD), and post-gastrointestinal (GI) surgery conditions (e.g., gastric bypass, ileal resection, biliary diversion) interfere with the uptake of essential fat-soluble (A, D, E, K) and water-soluble (e.g., B12) vitamins. These nutrients are critical for maintaining ocular surface integrity, phototransduction, optic nerve health, and retinal metabolism. Vitamin A deficiency, common in celiac and chronic pancreatitis patients, can result in xerophthalmia, Bitot’s spots, corneal ulceration, and night blindness [46].

Patients with vitamin B12 deficiency, particularly post-bariatric surgery or with ileal Crohn’s disease, are at increased risk for optic neuritis, bilateral optic atrophy, and central scotomas, often mimicking nutritional optic neuropathies seen in alcoholism [47]. Fat malabsorption in IBD and short bowel syndrome also leads to reduced uptake of carotenoids (lutein, zeaxanthin), which are critical for macular pigment density and prevention of age-related macular degeneration (AMD) [48].

Deficiencies of vitamins D and E are linked to ocular surface inflammation and delayed epithelial repair, suggesting that these micronutrients play complementary roles in maintaining corneal integrity and modulating local immune responses [49]. Although ocular findings related to vitamin K deficiency are rare, its role in regulating vascular calcification and maintaining endothelial stability may have indirect ocular implications. Low vitamin K status has been associated with increased retinal microvascular fragility and susceptibility to hemorrhage in systemic deficiency states. Experimental studies further suggest that vitamin K–dependent proteins, such as matrix Gla protein, contribute to retinal vascular homeostasis, indicating that adequate vitamin K intake may support ocular microcirculation [50]. Several studies have emphasized the importance of early ophthalmologic screening in patients with chronic gastrointestinal disease, particularly those with signs of malnutrition, poor weight gain, or long-term parenteral nutrition [51]. Prophylactic supplementation tailored to malabsorption etiology has shown benefits in preserving visual function and preventing irreversible retinal or optic nerve damage [35]. Reduced carotenoid bioavailability in malabsorption syndromes may also lead to lower macular pigment density, though evidence remains limited [35,51].

## 5. Nutritional Deficiencies in Special Populations

Certain population groups are more vulnerable to micronutrient deficiencies due to physiological, socioeconomic, or behavioral factors. Children in low- and middle-income countries are at high risk of vitamin A, iron, and zinc deficiencies due to inadequate weaning diets and high rates of infections. Ocular signs such as xerophthalmia, night blindness, Bitot’s spots, and conjunctival pallor are common and remain significant contributors to childhood morbidity and preventable blindness [52].

In the elderly population, nutritional risk increases due to factors such as reduced appetite, poor dentition, polypharmacy, gastrointestinal hypochlorhydria, and malabsorption syndromes. Deficiencies in vitamins D, E, C, and B12 are commonly reported and may manifest as dry eye disease, age-related macular degeneration (AMD), nuclear cataracts, and optic neuropathies. Multiple studies have linked subclinical malnutrition in aging adults to delayed recovery from ocular surgery, slower epithelial wound healing, and increased oxidative stress within retinal tissues [53,54].

Chronic alcoholics often suffer from B-complex vitamin deficiencies, especially thiamine (B1) and cobalamin (B12), due to impaired absorption, poor diet, and liver dysfunction. Clinically, this may present as nystagmus, gaze palsies, Wernicke’s encephalopathy, and nutritional optic neuropathy. Optic disc pallor, bilateral central scotomas, and loss of visual acuity are classic signs and often progress insidiously unless addressed early with parenteral supplementation [55,56]. Therefore, simple clinical tools such as dietary history, BMI assessment, and basic micronutrient testing may help identify individuals at higher nutritional risk.

## 6. Dietary Patterns, Obesity and Eye Health

Dietary patterns have a significant influence on ocular health, particularly through their modulation of oxidative stress, inflammation, and vascular function. The Mediterranean diet, characterized by high consumption of omega-3 fatty acids, leafy greens, olive oil, whole grains, nuts, and carotenoid-rich fruits and vegetables, has been consistently associated with a lower risk of age-related macular degeneration (AMD) and cataract progression [57]. High adherence to this diet has been shown to increase macular pigment optical density (MPOD) and preserve retinal function in aging populations [58].

In contrast, Western diets—often high in refined carbohydrates, saturated fats, and added sugars—are linked with an increased risk of AMD, diabetic retinopathy, and dry eye syndrome. High-glycemic index diets have been associated with increased glycation end-product formation and oxidative damage to retinal tissues, while low intake of lutein and zeaxanthin from leafy greens correlates with reduced macular protection [59,60].

Restrictive dietary patterns such as veganism, ketogenic, or very low-calorie diets may predispose individuals to vitamin B12, vitamin D, zinc, and iron deficiencies, especially without proper monitoring or supplementation. These micronutrient deficiencies are known risk factors for optic neuropathy, night blindness, and dry eye [61]. Clinical cases have been reported in long-term vegans with B12-related optic atrophy and in ketogenic dieters with selenium and magnesium imbalance affecting ocular function. Personalized nutrition counseling and dietary supplementation are essential when engaging in restrictive diets, particularly for patients with preexisting ocular conditions or genetic susceptibility to degenerative eye diseases [62,63].

Obesity represents a growing global health issue, closely linked to metabolic, vascular, and inflammatory alterations that also extend to ocular tissues. Excess adiposity contributes to systemic oxidative stress and endothelial dysfunction, both of which affect retinal microcirculation and the choroidal vasculature. Studies have shown that obesity is associated with a higher risk of diabetic retinopathy, AMD, and glaucoma, likely through mechanisms involving insulin resistance, dyslipidemia, and low-grade inflammation. In large epidemiological cohorts, obesity was associated with a 40% higher risk of diabetic retinopathy and a 30% higher likelihood of primary open-angle glaucoma [64]. In AMD, higher body mass index correlated inversely with macular pigment optical density, suggesting that adiposity may dilute carotenoid bioavailability and reduce retinal antioxidant defense [65].

Nutritional imbalance in obese individuals, often characterized by high caloric intake but low micronutrient density, can further exacerbate oxidative retinal damage and promote lipid peroxidation. Adipokines such as leptin and adiponectin are implicated in ocular pathophysiology, influencing angiogenic and inflammatory pathways relevant to retinal diseases [65]. Moreover, obesity-related sleep apnea and increased intracranial pressure are recognized contributors to papilledema and idiopathic intracranial hypertension, both of which can cause transient or permanent visual impairment [66]. Markers such as leptin, adiponectin, CRP, and oxidative stress biomarkers (e.g., MDA, 8-OHdG) may help clarify mechanistic links, although evidence remains limited and heterogeneous. Therefore, addressing obesity through dietary modulation, physical activity, and metabolic control plays a crucial role not only in systemic but also ocular health maintenance.

## 7. Nutritional Supplementation in AMD and Glaucoma: Evidence from Clinical Trials

The Age-Related Eye Disease Study (AREDS) demonstrated that high-dose antioxidant supplementation—including vitamin C (500 mg), vitamin E (400 IU), beta-carotene (15 mg), zinc (80 mg), and copper (2 mg)—reduced the progression of intermediate to advanced AMD by approximately 25% over 5 years [35]. Because beta-carotene was associated with increased lung-cancer risk in smokers, the AREDS2 trial replaced it with lutein (10 mg) and zeaxanthin (2 mg). This modified formula showed comparable or superior efficacy and improved macular pigment optical density, while avoiding the safety concerns associated with beta-carotene [67,68]. AREDS2 also evaluated omega-3 fatty acids, which did not provide additional benefit when added to the core formulation [67]. Overall, the AREDS/AREDS2 formulations remain the most robust evidence-based nutritional interventions for slowing progression in intermediate AMD, but they do not offer benefits in early disease [3].

A concise presentation summarizing the precise formulations of AREDS and AREDS2 is shown in Table 1.

In glaucoma, although antioxidant supplementation is not yet a standard therapy, magnesium and Ginkgo biloba extract (EGb761) have shown promise in improving ocular blood flow and protecting retinal ganglion cells. Magnesium acts as a calcium channel blocker and vascular smooth muscle relaxant, improving perfusion to the optic nerve head. Some small clinical studies have shown improvements in visual field stability and contrast sensitivity after magnesium supplementation [69]. Ginkgo biloba, through its vasodilatory and free radical-scavenging effects, has been associated with improved retinal circulation and visual field preservation, particularly in normal-tension glaucoma, though findings remain mixed and require larger trials for validation [70].

The main nutritional deficiencies and their associated ocular disorders are presented in Table 2. In addition, Table 3 provides an overview of the strength of evidence for the main nutritional–ocular associations.

## 8. Public Health Strategies and Screening Recommendations

Effective public health strategies are essential to address ocular manifestations of nutritional deficiencies at a population level. Key approaches include food fortification programs, such as the enrichment of cooking oils with vitamin A or iodized salt, which have significantly reduced xerophthalmia and night blindness in endemic regions [71]. Mass supplementation campaigns, particularly targeting children and postpartum women with vitamin A, iron, and folic acid, have also proven effective in lowering the prevalence of preventable visual impairments in low-resource settings [72].

Integrating eye health into primary care screening is crucial for early detection of micronutrient-related eye conditions. Regular ocular evaluations in high-risk groups such as preschool children, the elderly, and individuals with chronic illnesses or restrictive diets can identify early signs of dry eye, cataracts, or optic neuropathies before irreversible damage occurs/ In this context, nutritional screening and dietary assessment tools could be incorporated into ophthalmologic consultations, enhancing collaboration between public health, general practitioners, and eye care professionals [73].

Recent studies emphasize the importance of proactive screening for dry eye syndrome (DES) in patients with cardiovascular disease, where systemic medications and vascular factors can exacerbate ocular surface symptoms [74]. Early treatment, combined with dietary optimization, can significantly improve daily functioning, reinforcing the value of routine symptom-based screening even in non-ophthalmologic care settings [75].

Additionally, nutrient status evaluation should be considered an integral part of preventive ophthalmic care. Routine laboratory assessment of vitamin D, vitamin B12, zinc, and iron levels may be warranted, particularly among older adults, patients with malabsorption syndromes (e.g., inflammatory bowel disease or celiac disease), post-bariatric surgery patients, and individuals adhering to vegan diets. Periodic testing every 6–12 months allows early identification and correction of deficiencies before ocular manifestations appear. When necessary, supplementation should follow established recommended dietary allowance values (e.g., vitamin D 800–2000 IU/day, zinc 8–11 mg/day, vitamin B12 2.4 µg/day), unless otherwise guided by clinical context or laboratory findings [76,77].

Broader public health programs encouraging nutrient-rich dietary patterns—including green leafy vegetables, oily fish, eggs, and nuts, should be integrated into community eye health strategies. Educational interventions addressing UV protection, digital eye strain, and lifestyle modification can further promote ocular well-being and complement traditional ophthalmic care. Integrating ocular assessment into national nutritional screening programs may strengthen early detection and long-term prevention of deficiency-related eye diseases [76,77].

## 9. Clinical Implications and Recommendations

Clinicians should actively consider nutritional deficiencies in the differential diagnosis of unexplained ocular symptoms, particularly in cases of dry eye, optic neuropathies, and visual field defects where traditional etiologies are excluded. Routine nutritional screening is especially important in elderly patients, individuals with chronic illnesses, post-bariatric surgery patients, and those with restrictive diets, as these populations are at high risk for deficiencies in vitamins A, D, B12, and essential trace elements [78].

Dietary counseling, combined with laboratory monitoring, can facilitate the early detection and correction of deficiencies, helping to prevent irreversible ocular damage. Adequate vitamin D status plays a key role in maintaining ocular surface integrity and tear film stability, supporting epithelial regeneration and reducing inflammation [79].

Computer Vision Syndrome (CVS) represents a growing concern in modern ophthalmic practice due to prolonged screen exposure and reduced blink rate and is characterized by eye strain, blurred vision, and ocular surface instability [80]. Current evidence suggests that suboptimal micronutrient intake and obesity may exacerbate its symptoms, but direct nutritional causality remains limited. Therefore, this topic was briefly addressed, emphasizing ergonomic and preventive strategies alongside nutritional balance [81].

Evidence supports that targeted supplementation, including lutein, zeaxanthin, and antioxidants, contributes to retinal health, tear film stability, and neuroprotection, reducing progression of conditions such as AMD and dry eye disease [82].

### Clinical Implications—Practical Takeaways for Practitioners

- Nutritional deficiencies should be considered in the differential diagnosis of unexplained ocular symptoms such as chronic dry eye or optic neuropathies.
- Routine screening of vitamin D, B12, and zinc is recommended in elderly, vegan, and post-bariatric surgery patients.
- Laboratory monitoring and dietary counseling may prevent irreversible ocular damage through early correction of deficiencies.
- Balanced diets rich in carotenoids, omega-3 fatty acids, and antioxidants are preferable to isolated supplementation.
- Nutritional optimization and ergonomic measures can help reduce symptoms related to digital eye strain.

## 10. Conclusions

Current evidence shows that deficiencies in vitamins A, D, E, C, B-complex, and minerals such as zinc, selenium, and magnesium are consistently associated with major ocular diseases, including dry eye disease, cataracts, AMD, diabetic retinopathy, and optic neuropathies. Obesity also contributes to ocular morbidity through inflammation and metabolic imbalance. Overall, nutrients with the strongest support for ocular protection include antioxidant vitamins and trace elements, whereas findings for omega-3 fatty acids and dietary patterns remain variable.

Limitations: This review is narrative and includes studies with heterogeneous designs, populations, and nutritional assessment methods. Intermediate exclusion numbers were unavailable for all databases, and observational data are prone to confounding. Furthermore, variations in dietary reporting and biomarker thresholds limit direct comparisons between studies.

Future Perspectives: Future studies should include larger, well-controlled trials to clarify optimal nutrient combinations, dosing, and long-term effects on eye health. Standardized biomarkers for nutritional status are needed, as well as research exploring the interaction between diet, obesity, and ocular inflammation.

## Figures and Tables

**Table 1:** Composition of the AREDS and AREDS2 supplement formulations.

Component	AREDS (Original)	AREDS2 (Modified)
Vitamin C	500 mg	500 mg
Vitamin E	400 IU	400 IU
Beta-carotene	15 mg	replaced by lutein and zeaxanthin
Lutein	—	10 mg
Zeaxanthin	—	2 mg
Zinc	80 mg	80 mg
Copper	2 mg	2 mg
Omega-3	—	Evaluated separately—no added benefit
fatty acids		
(DHA + EPA)		

AREDS: Age-Related Eye Disease Study; AREDS2: Age-Related Eye Disease Study 2.

**Table 2:** Main nutritional deficiencies and their associated ocular disorders.

Nutrient	Associated Ocular Disease(s)	Author, Year	Main Evidence Type
Vitamin A	Xerophthalmia, night blindness, Bitot’s spots, keratomalacia	Faustino et al., 2016 [9]	Narrative review
Vitamin D	Dry eye disease, posterior subcapsular cataract, diabetic retinopathy	Rolando et al., 2023 [14] Bhatt et al., 2023 [15]	Review on DED Observational
Vitamin E	Retinopathy, decreased contrast sensitivity, AMD progression	Christen et al., 2010 [17] Traber, 2014 [18]	Randomized controlled trial Review
Vitamin B1 (Thiamine)	Wernicke’s encephalopathy (nystagmus, ophthalmoplegia)	Sechi et al., 2007 [21]	Review
Vitamin B2 (Riboflavin)	Keratoconjunctivitis, photophobia	Powers, 2003 [22]	Review
Vitamin B6	Optic neuropathy (bilateral vision loss, central scotomas)	Roda et al., 2020 [23]	Review
Vitamin B12	Optic neuropathy, visual field loss	Roda et al., 2020 [23] Gu et al., 2023 [24]	Review Review
Folate (B9)	Retinal metabolism disruption, vascular disease	Gu et al., 2023 [24]	Review
Vitamin C	Subconjunctival hemorrhage, delayed healing, cataracts	Chambial et al., 2013 [25] Moser et al., 2016 [27]	Review Review
Zinc	Night blindness, delayed healing, AMD	Gilbert et al., 2019 [33] AREDS, 2001 [19]	Review Randomized controlled trial
Copper	Optic neuropathy, bilateral blindness, myelopathy	Naismith et al., 2009 [37] Amin et al., 2022 [39]	Case report/case series Case report
Selenium	Cataracts, retinal degeneration, diabetic retinopathy	Chen et al., 2025 [40] Xu et al., 2023 [41] Al-Bassam et al., 2024 [42]	Observational Observational cohort Review
Magnesium	Glaucoma, diabetic retinopathy	Ekici et al., 2014 [44] Elghobashy et al., 2022 [45]	Review Review
Vitamin K	Retinal hemorrhages, ocular inflammation (rare)	Mong, 2023 [50]	Review
Carotenoids	AMD, cataracts, visual performance loss	Khoo et al., 2019 [35]	Review

Abbreviations: AMD = age-related macular degeneration.

**Table 3:** Summary Table of Evidence Strength.

Nutritional Factor	Ocular Condition	Main Evidence Type	Strength of Evidence
AREDS/AREDS2 antioxidant formulation	AMD progression	RCTs, meta-analyses	High
Vitamin D	Dry eye disease, AMD	Cross-sectional, small RCTs	Moderate
Obesity	Diabetic retinopathy, AMD, glaucoma	Cohort studies	Moderate
Magnesium	Glaucoma, ocular blood flow	Observational, pilot trials	Low–Moderate
Omega-3 fatty acids	Dry eye, AMD	Mixed interventional trials	Moderate

Evidence strength was assessed qualitatively according to study design and consistency (high: supported by RCTs/meta-analyses; moderate: supported by cohort/case–control studies; low: based on cross-sectional or mechanistic data). Abbreviations: AMD = age-related macular degeneration; RCT = randomized controlled trial; AREDS = Age-Related Eye Disease Study; AREDS2 = Age-Related Eye Disease Study 2.

## Data Availability

Data sharing is not applicable to this article.

## References

[1] Beatty S., Koh H., Phil M., Henson D., Boulton M. (2000). The role of oxidative stress in the pathogenesis of age-related macular degeneration. Surv. Ophthalmol..

[2] Pellegrini M., Senni C., Bernabei F., Cicero A.F.G., Vagge A., Maestri A., Scorcia V., Giannaccare G. (2020). The Role of Nutrition and Nutritional Supplements in Ocular Surface Diseases. Nutrients.

[3] Evans J.R., Lawrenson J.G. (2023). Antioxidant vitamin and mineral supplements for slowing the progression of age-related macular degeneration. Cochrane Database Syst. Rev..

[4] Huang Z., Liu S., Chen C., Zhang K., Du Y., Zhu X. (2025). Optimizing serum 25(OH)D levels to mitigate the risk of age-related ocular diseases: Insights from a large-scale prospective cohort study. Nutr. J..

[5] Luo X., Ruan Z., Liu L. (2025). Causal effect of the 25-Hydroxyvitamin D concentration on ocular diseases: A Mendelian randomization study. Sci. Rep..

[6] Pieńczykowska K., Bryl A., Mrugacz M. (2025). The Impact of a High-Fat Diet on Eye Health. Nutrients.

[7] Marques-Couto P., Coelho-Costa I., Ferreira-da-Silva R., Andrade J.P., Carneiro Â. (2025). Mediterranean Diet on Development and Progression of Age-Related Macular Degeneration: Systematic Review and Meta-Analysis of Observational Studies. Nutrients.

[8] Page M.J., McKenzie J.E., Bossuyt P.M., Boutron I., Hoffmann C.T., Mulrow C.D., Shamseer L., Tetzlaff J.M., Akl E.A., Brennan S.E. (2021). The PRISMA 2020 statement: An updated guideline for reporting systematic reviews. BMJ..

[9] Faustino J.F., Ribeiro-Silva A., Dalto R.F., Souza M.M., Furtado J.M., Rocha G.deM., Alves M., Rocha E.M. (2016). Vitamin A and the eye: An old tale for modern times. Arq. Bras. Oftalmol..

[10] Stevens G.A., Bennett J.E., Hennocq Q., Lu Y., De-Regil L.M., Rogers L., Danaei G., Li G., White R.A., Flaxman S.R. (2015). Trends and mortality effects of vitamin A deficiency in children in 138 low-income and middle-income countries. Lancet Glob. Health.

[11] Wiseman E.M., Bar-El Dadon S., Reifen R. (2017). The vicious cycle of vitamin A deficiency: A review. Crit. Rev. Food Sci. Nutr..

[12] Hyon J.Y., Han S.B. (2022). Dry Eye Disease and Vitamins: A Narrative Literature Review. Appl. Sci..

[13] Wirth J.P., Petry N., Tanumihardjo S.A., Rogers L.M., McLean E., Greig A., Garrett G.S., Klemm R.D., Rohner F. (2017). Vitamin A Supplementation Programs and Country-Level Evidence of Vitamin A Deficiency. Nutrients.

[14] Rolando M., Barabino S. (2023). Dry Eye Disease: What Is the Role of Vitamin D?. Int. J. Mol. Sci..

[15] Bhatt R.B., Patel N.H., Shah A.T., Ranpara K.H. (2023). Study of correlation between vitamin D3 levels and dry eye. Indian. J. Ophthalmol..

[16] Gverović Antunica A., Znaor L., Ivanković M., Puzović V., Marković I., Kaštelan S. (2023). Vitamin D and Diabetic Retinopathy. Int. J. Mol. Sci..

[17] Christen W.G., Glynn R.J., Chew E.Y., Buring J.E. (2010). Vitamin E and age-related macular degeneration in a randomized trial of women. Ophthalmology.

[18] Traber M.G. (2014). Vitamin E inadequacy in humans: Causes and consequences. Adv. Nutr..

[19] Age-Related Eye Disease Study Research Group (2001). A randomized, placebo-controlled, clinical trial of high-dose supplementation with vitamins C and E, beta-carotene, and zinc for age-related macular degeneration and vision loss: AREDS report no. 8. Arch. Ophthalmol..

[20] Garcia-Medina J.J., Rubio-Velazquez E., Foulquie-Moreno E., Casaroli-Marano R.P., Pinazo-Duran M.D., Zanon-Moreno V., Del-Rio-Vellosillo M. (2020). Update on the Effects of Antioxidants on Diabetic Retinopathy: In Vitro Experiments, Animal Studies and Clinical Trials. Antioxidants.

[21] Sechi G., Serra A. (2007). Wernicke’s encephalopathy: New clinical settings and recent advances in diagnosis and management. Lancet Neurol..

[22] Powers H.J. (2003). Riboflavin (vitamin B2) and health. Am. J. Clin. Nutr..

[23] Roda M., di Geronimo N., Pellegrini M., Schiavi C. (2020). Nutritional Optic Neuropathies: State of the Art and Emerging Evidences. Nutrients.

[24] Gu J., Lei C., Zhang M. (2023). Folate and retinal vascular diseases. BMC Ophthalmol..

[25] Chambial S., Dwivedi S., Shukla K.K., John P.J., Sharma P. (2013). Vitamin C in disease prevention and cure: An overview. Indian. J. Clin. Biochem..

[26] Mandl J., Szarka A., Bánhegyi G. (2009). Vitamin C: Update on physiology and pharmacology. Br. J. Pharmacol..

[27] Moser M.A., Chun O.K. (2016). Vitamin C and eye health: Epidemiologic evidence. Nutrients.

[28] De Tullio M.C. (2012). Beyond the antioxidant: The double life of vitamin C. Subcell. Biochem..

[29] Kulbay M., Wu K.Y., Nirwal G.K., Bélanger P., Tran S.D. (2024). Oxidative Stress and Cataract Formation: Evaluating the Efficacy of Antioxidant Therapies. Biomolecules.

[30] Hah Y.S., Chung H.J., Sontakke S.B., Chung I.Y., Ju S., Seo S.W., Yoo J.M., Kim S.J. (2017). Ascorbic acid concentrations in aqueous humor after systemic vitamin C supplementation in patients with cataract: Pilot study. BMC Ophthalmol..

[31] Lou M.F. (2003). Redox regulation in the lens. Prog. Retin. Eye Res..

[32] Chen J., Lan J., Liu D., Backman L.J., Zhang W., Zhou Q., Danielson P. (2017). Ascorbic Acid Promotes the Stemness of Corneal Epithelial Stem/Progenitor Cells and Accelerates Epithelial Wound Healing in the Cornea. Stem Cells Transl. Med..

[33] Gilbert R., Peto T., Lengyel I., Emri E. (2019). Zinc Nutrition and Inflammation in the Aging Retina. Mol. Nutr. Food Res..

[34] Serhan H.A., Alma’aitah H.W., Irshaidat S., Ameer M.A., Asghar M.S., Tahir M.J. (2022). Ophthalmic manifestations of nutritional deficiencies: A mini review. J. Family Med. Prim. Care.

[35] Khoo H.E., Ng H.S., Yap W.S., Goh H.J.H., Yim H.S. (2019). Nutrients for Prevention of Macular Degeneration and Eye-Related Diseases. Antioxidants.

[36] Blasiak J., Pawlowska E., Chojnacki J., Szczepanska J., Chojnacki C., Kaarniranta K. (2020). Zinc and Autophagy in Age-Related Macular Degeneration. Int. J. Mol. Sci..

[37] Naismith R.T., Shepherd J.B., Weihl C.C., Tutlam N.T., Cross A.H. (2009). Acute and bilateral blindness due to optic neuropathy associated with copper deficiency. Arch. Neurol..

[38] Jaiser S.R., Winston G.P. (2010). Copper deficiency myelopathy. J. Neurol..

[39] Amin A., Khoury N.C., Lacayo M., Kostanyan S. (2022). Copper Deficiency-Induced Neuropathy After Bariatric Surgery Disguised as Demyelinating Disease: A Case Report. Cureus.

[40] Chen X., Sun M., Gu Z., Hao X., Xie L. (2025). Association of serum selenium levels with diabetic retinopathy: NHANES 2011-2016. Front. Med..

[41] Xu B., Liu Z., Zhao J., Yu Z. (2023). Selenium intake help prevent age-related cataract formation: Evidence from NHANES 2001-2008. Front. Nutr..

[42] Al-Bassam L., Shearman G.C., Brocchini S., Alany R.G., Williams G.R. (2024). The Potential of Selenium-Based Therapies for Ocular Oxidative Stress. Pharmaceutics.

[43] Shivakumar K., Rajalakshmi A.R., Jha K.N., Nagarajan S., Srinivasan A.R., Lokesh Maran A. (2021). Serum magnesium in diabetic retinopathy: The association needs investigation. Ther. Adv. Ophthalmol..

[44] Ekici F., Korkmaz Ş., Karaca E.E., Sül S., Tufan H.A., Aydın B., Dileköz E. (2014). The Role of Magnesium in the Pathogenesis and Treatment of Glaucoma. Int. Sch. Res. Notices.

[45] Elghobashy M., Lamont H.C., Morelli-Batters A., Masood I., Hill L.J. (2022). Magnesium and Its Role in Primary Open Angle Glaucoma; A Novel Therapeutic?. Front. Ophthalmol..

[46] Scarampi M., Mengoli C., Miceli E., Di Stefano M. (2025). Vitamins and Celiac Disease: Beyond Vitamin D. Metabolites.

[47] Sawicka-Pierko A., Obuchowska I., Hady R.H., Mariak Z., Dadan J. (2014). Nutritional optic neuropathy following bariatric surgery. Wideochir Inne Tech. Maloinwazyjne.

[48] Ward M.S., Zhao D.Y., Bernstein P.S. (2008). Macular and serum carotenoid concentrations in patients with malabsorption syndromes. J. Ocul. Biol. Dis. Infor..

[49] Reins R.Y., McDermott A.M. (2015). Vitamin D: Implications for ocular disease and therapeutic potential. Exp. Eye Res..

[50] Mong M.A. (2023). Vitamin K and the Visual System-A Narrative Review. Nutrients.

[51] Troncoso L.L., Biancardi A.L., de Moraes H.V., Zaltman C. (2017). Ophthalmic manifestations in patients with inflammatory bowel disease: A review. World J. Gastroenterol..

[52] Akhtar S., Ahmed A., Randhawa M.A., Atukorala S., Arlappa N., Ismail T., Ali Z. (2013). Prevalence of vitamin A deficiency in South Asia: Causes, outcomes, and possible remedies. J. Health Popul. Nutr..

[53] Braakhuis A., Raman R., Vaghefi E. (2017). The Association between Dietary Intake of Antioxidants and Ocular Disease. Diseases.

[54] Hickson M. (2006). Malnutrition and ageing. Postgrad. Med. J..

[55] Liberski S., Kaluzny B.J., Kocięcki J. (2022). Methanol-induced optic neuropathy: A still-present problem. Arch. Toxicol..

[56] Isen D.R., Kline L.B. (2020). Neuro-ophthalmic Manifestations of Wernicke Encephalopathy. Eye Brain.

[57] Wu Y., Xie Y., Yuan Y., Xiong R., Hu Y., Ning K., Ha J., Wang W., Han X., He M. (2023). The Mediterranean Diet and Age-Related Eye Diseases: A Systematic Review. Nutrients.

[58] Gastaldello A., Giampieri F., Quiles J.L., Navarro-Hortal M.D., Aparicio S., García Villena E., Tutusaus Pifarre K., De Giuseppe R., Grosso G., Cianciosi D. (2022). Adherence to the Mediterranean-Style Eating Pattern and Macular Degeneration: A Systematic Review of Observational Studies. Nutrients.

[59] Chiu C.J., Milton R.C., Gensler G., Taylor A. (2007). Association between dietary glycemic index and age-related macular degeneration in nondiabetic participants in the Age-Related Eye Disease Study. Am. J. Clin. Nutr..

[60] Masri A., Armanazi M., Inouye K., Geierhart D.L., Davey P.G., Vasudevan B. (2024). Macular Pigment Optical Density as a Measurable Modifiable Clinical Biomarker. Nutrients.

[61] Bali A., Naik R. (2023). The Impact of a Vegan Diet on Many Aspects of Health: The Overlooked Side of Veganism. Cureus.

[62] Teng R.W., Heidary G., Gise R.A. (2024). Selective diet induced nutritional optic neuropathy in developmentally normal children. Am. J. Ophthalmol. Case Rep..

[63] Crosby L., Davis B., Joshi S., Jardine M., Paul J., Neola M., Barnard N.D. (2021). Ketogenic Diets and Chronic Disease: Weighing the Benefits Against the Risks. Front. Nutr..

[64] Cheung N., Wong T.Y. (2007). Obesity and eye diseases. Surv. Ophthalmol..

[65] Almpanidou S., Vachliotis I.D., Goulas A., Polyzos S.A. (2025). The potential role of adipokines and hepatokines in age-related ocular diseases. Metab. Open.

[66] Andrews L.E., Liu G.T., Ko M.W. (2014). Idiopathic intracranial hypertension and obesity. Horm. Res. Paediatr..

[67] AREDS2 Research Group (2013). Lutein + zeaxanthin and omega-3 fatty acids for age-related macular degeneration: The AREDS2 randomized clinical trial. JAMA.

[68] Carneiro Â., Andrade J.P. (2017). Nutritional and Lifestyle Interventions for Age-Related Macular Degeneration: A Review. Oxid. Med. Cell Longev..

[69] Aydin B., Onol M., Hondur A., Kaya M.G., Ozdemir H., Cengel A., Hasanreisoglu B. (2010). The effect of oral magnesium therapy on visual field and ocular blood flow in normotensive glaucoma. Eur. J. Ophthalmol..

[70] Cybulska-Heinrich A.K., Mozaffarieh M., Flammer J. (2012). Ginkgo biloba: An adjuvant therapy for progressive normal and high tension glaucoma. Mol. Vis..

[71] West K.P. (2003). Vitamin A deficiency disorders in children and women. Food Nutr. Bull..

[72] Song P., Adeloye D., Li S., Zhao D., Ye X., Pan Q., Qiu Y., Zhang R., Rudan I., Global Health Epidemiology Research Group (GHERG) (2023). The prevalence of vitamin A deficiency and its public health significance in children in low- and middle-income countries: A systematic review and modelling analysis. J. Glob. Health..

[73] Flaxman S.R., Bourne R.R.A., Resnikoff S., Ackland P., Braithwaite T., Cicinelli M.V., Das A., Jonas J.B., Keeffe J., Kempen J.H. (2017). Global causes of blindness and distance vision impairment 1990-2020: A systematic review and meta-analysis. Lancet Glob. Health.

[74] Martinescu G., Bogdanici C.M., Niagu I.A., Ciocoiu M. (2021). Dry eye syndrome in patients with cardiovascular pathology. Med.-Surg. J. Rev. Med. Chir. Soc. Med. Nat..

[75] Martinescu G., Bogdanici C.M., Pavel I.A., Ciocoiu M. (2022). Difficulties in performing daily activities in patients with dry eye before and after treatment. Medicina.

[76] Thirunavukkarasu A., Alanazi B., Alfaleh A., Alsulami H.H., Albudayr S.A., Alotaibi A.S., Alenezi R.M., Alruwaili A.G., Alibrahim N.O. (2024). Evaluation of dietary patterns and their impact on eye health among Saudi adults-A multi-regional cross-sectional analysis in Makkah, Riyadh, and Qassim. Front. Nutr..

[77] Murkey S.P., Agarwal A., Pandit P., Kumar S., Jaiswal A. (2023). Unveiling the Spectrum of Ophthalmic Manifestations in Nutritional Deficiencies: A Comprehensive Review. Cureus.

[78] Allen L.H. (2008). Causes of vitamin B12 and folate deficiency. Food Nutr. Bull..

[79] Mrugacz M., Pieńczykowska K., Bryl A. (2024). The Role of Vitamin D3 in Ocular Diseases. Nutrients.

[80] Pavel I.A., Andronic D.G., Bogdanici C.M. (2022). Computer Vision Syndrome in Medical Students and Healthcare Workers. Med.-Surg. J. Rev. Med. Chir. Soc. Med. Nat..

[81] Lem D.W., Gierhart D.L., Davey P.G. (2022). Can Nutrition Play a Role in Ameliorating Digital Eye Strain?. Nutrients.

[82] Hobbs R.P., Bernstein P.S. (2014). Nutrient Supplementation for Age-related Macular Degeneration, Cataract, and Dry Eye. J. Ophthalmic Vis. Res..

